# The impact of phosphodiesterase‐5 inhibition or angiotensin‐converting enzyme inhibition on right and left ventricular remodeling in heart failure due to chronic volume overload

**DOI:** 10.1002/prp2.1172

**Published:** 2024-01-29

**Authors:** Tereza Tykvartova, Matus Miklovic, Martin Kotrc, Petra Skaroupkova, Ludmila Kazdova, Jaroslava Trnovska, Vojtech Skop, Michal Kolar, Jiri Novotny, Vojtech Melenovsky

**Affiliations:** ^1^ Institute for Clinical and Experimental Medicine—IKEM Prague Czech Republic; ^2^ Department of Pathophysiology, Second Faculty of Medicine Charles University Prague Czech Republic; ^3^ Department of Biochemistry and Microbiology University of Chemistry and Technology Prague Czech Republic; ^4^ Laboratory of Genomics and Bioinformatics Institute of Molecular Genetics of the Czech Academy of Sciences Prague Czech Republic

**Keywords:** heart failure, phosphodiesterase‐5 inhibition, rats, right ventricle, volume overload

## Abstract

While phosphodiesterase‐5 inhibition (PED5i) may prevent hypertrophy and failure in pressure‐overloaded heart in an experimental model, the impact of PDE5i on volume‐overload (VO)‐induced hypertrophy is unknown. It is also unclear whether the hypertrophied right ventricle (RV) and left ventricle (LV) differ in their responsiveness to long‐term PDE5i and if this therapy affects renal function. The goal of this study was to elucidate the effect of PDE5i treatment in VO due to aorto‐caval fistula (ACF) and to compare PDE5i treatment with standard heart failure (HF) therapy with angiotensin‐converting enzyme inhibitor (ACEi). ACF/sham procedure was performed on male HanSD rats aged 8 weeks. ACF animals were randomized for PDE5i sildenafil, ACEi trandolapril, or placebo treatments. After 20 weeks, RV and LV function (echocardiography, pressure‐volume analysis), myocardial gene expression, and renal function were studied. Separate rat cohorts served for survival analysis. ACF led to biventricular eccentric hypertrophy (LV: +68%, RV: +145%), increased stroke work (LV: 3.6‐fold, RV: 6.7‐fold), and reduced load‐independent systolic function (PRSW, LV: −54%, RV: −51%). Both ACF ventricles exhibited upregulation of the genes of myocardial stress and glucose metabolism. ACEi but not PDE5i attenuated pulmonary congestion, LV remodeling, albuminuria, and improved survival (median survival in ACF/ACEi was 41 weeks vs. 35 weeks in ACF/placebo, *p* = .02). PDE5i increased cyclic guanosine monophosphate levels in the lungs, but not in the RV, LV, or kidney. PDE5i did not improve survival rate and cardiac and renal function in ACF rats, in contrast to ACEi. VO‐induced HF is not responsive to PDE5i therapy.

## Graphical abstract

Despite elevated cGMP levels in the lungs of sildenafil‐treated rats, PDE5i did not improve survival rate and did not change the cardiac and renal function of ACF rats with advanced HF. In contrast, mortality in the ACF model was reduced by ACEi treatment.

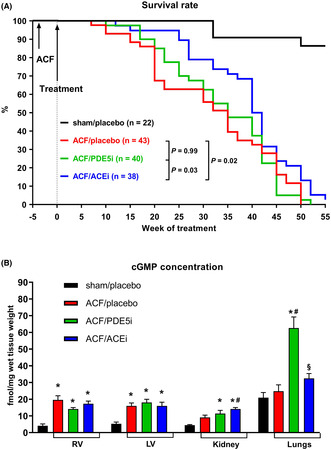

**Abbreviations**
ACEi: angiotensin‐converting enzyme inhibitor
ACF: aorto‐caval fistula
DBP: diastolic blood pressure
dP/dt max: maximum ventricular pressure over time
dP/dt min: minimum ventricular pressure over time
EDP: ventricular end‐diastolic pressure
EDV: end‐diastolic volume
ESV: end‐systolic volume
FAC: fractional area change
HF: heart failure
HanSD: Hannover Sprague Dawley rats
LV: left ventricle
MBP: mean blood pressure
PDE5: phosphodiesterase 5
PDE9: phosphodiesterase 9
PED5i: phosphodiesterase‐5 inhibition
PP: pulse pressure
PRSW: preload recruitable stroke work
RAAS: renin‐angiotensin‐aldosterone system
RV: right ventricle
SBP: systolic blood pressure
SVR: systemic vascular resistance
TAPSE: tricuspid annular plane systolic excursion
Tau: ventricular diastolic time constant
VO: volume‐overload

## 1 INTRODUCTION

Heart failure (HF) is a progressive clinical syndrome with high morbidity, mortality, and constantly increasing prevalence that imposes a great burden on healthcare systems worldwide. It is crucial to develop novel therapeutic strategies to prevent or stabilize the course of the disease. Right ventricular (RV) dysfunction and renal dysfunction accelerate HF progression and are associated with increased HF mortality. However, detailed pathogenic mechanisms of both these conditions and their role in HF remain incompletely understood.

The aorto‐caval fistula (ACF) represents a well‐defined model to study advanced HF, characterized by eccentric cardiac hypertrophy and dysfunction, neurohormonal alterations including activation of the renin‐angiotensin‐aldosterone system (RAAS), congestion, and renal function impairment.^1^, ^2^, ^3^, ^4^ Blood recirculation via ACF imposes the same volume overload (VO) on the left and right heart, which makes the model useful for studying biventricular differences in response to experimental therapies.^5^, ^6^

RAAS activation is antagonized by nitric oxide and natriuretic peptides signaling, two systems that stimulate guanylate cyclase to produce cyclic guanosine monophosphate (cGMP). The concentration of cGMP is strictly regulated and catalyzed by phosphodiesterases including phosphodiesterase 5 (PDE5) and 9 (PDE9), which represent potential pharmacological targets for the treatment of chronic HF.^7^

Although it is not significantly expressed in healthy heart, PDE5 overexpression was demonstrated in hypertrophied or failing myocardium of both RV and left ventricle (LV).^8^, ^9^, ^10^ Apart from its vasodilatory properties, used in clinical practice to treat pulmonary hypertension or erectile dysfunction, PDE5 inhibitor (PDE5i) sildenafil was shown to have anti‐hypertrophic and anti‐apoptotic effects on isolated cardiac myocytes.^11^, ^12^ In experimental studies, sildenafil prevented and reversed hypertrophy and dysfunction of pressure‐overloaded LV,^13^ attenuated fibrosis, decreased pulmonary pressure, and enhanced both systolic and diastolic function of pressure‐overloaded RV.^14^, ^15^, ^16^ Whether these findings are applicable to hypertrophy induced by VO is uncertain. Signaling pathways involved in the pressure versus VO‐induced hypertrophy differ considerably, and these pathological states may therefore require different pharmacotherapeutic interventions.^17^, ^18^ The impact of these interventions may vary between RV and LV,^19^ but biventricular differences are rarely addressed.

In experimental HF, there is a notable upregulation of PDE5 in the kidney,^20^ suggesting that PDE5i may have beneficial renal effects in HF.^21^, ^22^ The aim of this study was to evaluate the effect of long‐term PDE5i on cardiac and renal function and survival in the rat ACF model and to identify potential differences in PDE5i effects on volume‐overloaded LV or RV.

This task was addressed using load‐independent biventricular pressure‐volume analysis, echocardiography, assessment of renal hemodynamics and excretory function, and myocardial gene expression analysis of selected genes associated with HF development,^23^, ^24^ including the genes of the cGMP‐dependent signaling pathway.^25^, ^26^ The effect of PDE5i on VO‐induced HF was compared to an angiotensin‐converting enzyme inhibitor (ACEi), which represents a current therapeutic standard of HF.^27^, ^28^

## 2 MATERIALS AND METHODS

### 2.1 HF model

Male Hannover Sprague Dawley rats (HanSD) aged 8 weeks weighing 280–320 g and derived from an on‐site certified breeding colony at IKEM were randomly assigned to two groups and underwent needle ACF or sham operation as described before.^4^, ^29^ Briefly, the rats were anesthetized with the ketamine/midazolam mixture (Calypsol, Gedeon Richter, Hungary, 160 mg/kg and Midazolam, Kalcex, Latvia, 160 mg/kg, i.p.) and a shunt was created between the infrarenal aorta and inferior vena cava using an 18‐gauge needle (outer diameter 1.2 mm). The puncture site in the aorta was closed with acrylamide tissue glue (Histoacryl, B. Braun AG, Germany). The rats were housed in an air‐conditioned animal facility on a 12/12‐h light/dark cycle and were fed a standard salt/protein chow (0.45% NaCl, 19%–21% protein, SEMED, Czech Republic) with free access to tap water. Rats were weighed every week and the development of HF was assessed by the scoring procedure as described before.^3^ At the end of the protocol, the creation of ACF was confirmed by laparotomy and was found successful in each case. The rats were exsanguinated, and the coronary arteries of the explanted heart were rapidly infused with a cardioplegic solution. The organs were weighed, and the weight values were factored by body weight. The investigation was performed in accordance with the NIH Guide for the Care and Use of Laboratory Animals (NIH Publication No. 85‐23, 1996) and Animal protection laws of the Czech Republic (311/1997) and was approved by the Ethic Committee of IKEM.

### 2.2 Study design

Four weeks after ACF creation, ACF animals were randomly assigned to treatment groups with PDE5i sildenafil (Vizarsin, Krka, Slovenia, 80 mg/kg/day dissolved in drinking water, *N* = 76), ACEi trandolapril (Gopten, Mylan, Ireland, 6 mg/L dissolved in drinking water, *N* = 84), or placebo (*N* = 87). After 20 weeks, echocardiography and biventricular pressure‐volume analysis (*N* = 19–30/group), myocardial gene expression analysis (qPCR, *N* = 12–13/group), renal hemodynamic studies (*N* = 13–16/group), and cGMP tissue level measurements (*N* = 8/group) were performed as described below. A separate rat cohort (*N* = 22‐43/group) served for survival analysis and assessment of albuminuria.

### 2.3 Echocardiography and hemodynamics

Echocardiography was performed under general anesthesia (ketamine/midazolam mixture given intraperitoneally as described above) using a 10 MHz transducer (Vivid System 7 Dimension; GE HealthCare, IL, USA). RV fractional area change (FAC) was defined as a difference between end‐diastolic and end‐systolic RV area, divided by end‐diastolic area. RV volumes were calculated by the monoplane ellipsoid approximation method.^30^ Subsequently, ventricular function was invasively assessed with 2F Pressure–Volume micromanometer‐tip catheters (Millar, Houston, TX, USA) simultaneously introduced into the LV via the right carotid artery and into the RV via the internal jugular vein,^6^ which is considered “gold standard” in the evaluation of systolic and diastolic function of the LV and RV.^31^ The volume signals were adjusted by end‐diastolic (EDV) and end‐systolic volumes (ESV) gained by echocardiography shortly before invasive recordings as described previously.^29^ The data were obtained using an 8‐channel Power Lab recorder and analyzed by Labchart Pro software (ADInstruments, Bella Vista, NSW, Australia).

### 2.4 Myocardial gene expression analysis

Samples of RV and LV free wall were placed into RNA later, total RNA was isolated, and genomic DNA was removed. RNA quantity and integrity were measured. The RNA was reverse transcribed and qPCR was performed employing RealTime ready Custom Panel 384–32 (Roche, p.n. 05582962001) containing function tested pre‐plated qPCR assays for 29 target genes (*Acadm*, *Acta1*, *Angpt1*, *Angpt2*, *Apln*, *Aplnr*, *Atp2a2*, *Cs*, *Gucy1a3*, *Hif1a*, *Hk1*, *Il6*, *Maoa*, *Myh6*, *Myh7*, *Nos1*, *Nos2*, *Nos3*, *Nppa*, *Npr1*, *Npr2*, *Pde5a*, *Pde9a*, *Pkg*, *Slc2a1*, *Slc2a4*, *Tgm2*, *Tnfrsf1a*, *Vegfa*) and 3 reference genes average (*Hprt1*, *Sdha*, *Tbp*). The analysis was done on a LightCycler LC480 (Roche) according to the manufacturer's protocol. The acquired data were analyzed by the ∆Cp method within the R/Bioconductor statistical environment.^32^, ^33^, ^34^ The expression of mRNA of selected genes was related to a control group. The final results were expressed as the fold change difference of mRNA of the target gene between the experimental and control group.

### 2.5 Renal hemodynamics and excretory function

Renal hemodynamic studies were performed according to the previously described protocol.^29^, ^35^ Briefly, the animals were anesthetized with thiopental sodium (50 mg/kg, i.p., VAUB Pharma a.s., Roztoky, CZ), and the left femoral artery was cannulated to measure arterial blood pressure. The left kidney was surgically isolated from the surrounding tissues and put in a lucite cup. The left ureter was catheterized to collect urine, and an ultrasonic transient‐time flow probe (1RBF, Transonic Systems, Altron Medical Electronic GmbH, Germany) was installed on the left renal artery for continuous measurement of renal blood flow. A 0.5 mL bolus of 5% sinistrin (Inutest, Fresenius Kabi Austria GmbH, Austria) was administered to measure the glomerular filtration rate. Urine was gathered in three 30‐min periods and blood samples were obtained after each collection. Urine volume was measured by gravimetry; sodium and potassium concentrations were determined photometrically. Values were expressed per gram of wet kidney weight. The fractional sodium and potassium excretion were calculated by standard formulas.

### 2.6 Albuminuria

For albumin excretion measurement, the rats were placed in individual metabolic cages and (after appropriate habituation training) the urine was collected for 24 h in week 1, 4, 12, 20, 24, 28, 32, and 44 after initiation of treatment. Urinary albumin was measured using the quantitative sandwich enzyme immunoassay technique with the commercially available ELISA kit (ERA3201‐1, AssayPro, MO, USA).

### 2.7 cGMP tissue concentrations

All analyses were performed with the acetylation protocol to achieve maximal sensitivity. Briefly, tissue samples frozen in liquid nitrogen were crushed to a fine powder and homogenized in 0.1 M HCl. Precipitated proteins were separated by centrifugation at 20 000 *g* at 4°C. cGMP levels were measured in acetylated supernatants using a radioimmunoassay kit (cGMP‐RIA) from IBL International (GmbH, Hamburg, Germany) according to the manufacturer's protocol. Results are shown as fmol cGMP per milligram of wet tissue weight.

### 2.8 Statistical analysis

Statistical analysis was performed using Graph‐Pad Prism software v9.4.1 (Graph Pad Software, San Diego, CA, USA). The groups were compared by one‐way ANOVA and Tukey post hoc tests. The comparison of survival curves was performed using the log‐rank (Mantel‐Cox) test followed by Gehan‐Breslow‐Wilcoxon test. Results are presented as means ± SD, if not stated otherwise. A *p*‐value lower than .05 was considered significant.

### 2.9 Nomenclature of targets and ligands

Key protein targets and ligands in this article are hyperlinked to corresponding entries in http://www.guidetopharmacology.org, the common portal for data from the IUPHAR/BPS Guide to Pharmacology^36^ and are permanently archived in the Concise Guide to Pharmacology 2019/20.^37^

## 3 RESULTS

### 3.1 Organ weights and signs of HF

Twenty‐four weeks after ACF creation, 70% of ACF rats exhibited clinical signs of HF. ACF animals had similar tibial length but the body weight was increased (+10%, *p* < .01), likely due to congestion (Table 1; Supporting Information 1). ACF led to significant biventricular hypertrophy, more pronounced for the RV than for the LV (+145%, *p* < .001 and + 68%, *p* < .001), to increased lung weight (+41%, *p* < .001) due to congestion, and to reduced kidney weight (−17%, *p* < .001), probably related to hypoperfusion. ACEi treatment lowered body weight (−9%, *p* < .01) and lung weight (−14%, *p* < .05), indicating reduced congestion. PDE5i treatment did not alter the body and organ weight values (Table 1).

**TABLE 1:** Baseline characteristics and echocardiography.

	Sham/placebo	ACF/placebo	ACF/PDE5i	ACF/ACEi	*p* (ANOVA)
Body weight (BW), g	550 ± 47	607 ± 71**	584 ± 45	550 ± 50^##^	.0006
Tibial length, mm	43.1 ± 1.08	43.5 ± 1.42	43 ± 1.77	43.2 ± 1.09	.6
Heart weight/BW, g kg^−1^	2.97 ± 0.24	5.92 ± 0.77***	6 ± 0.69***	5.53 ± 0.86***	<.0001
LV weight/BW, g kg^−1^	2.03 ± 0.17	3.42 ± 0.45***	3.54 ± 0.39***	3.15 ± 0.38***^§§^	<.0001
RV weight/BW, g kg^−1^	0.53 ± 0.05	1.3 ± 0.23***	1.33 ± 0.21***	1.22 ± 0.18***	<.0001
Atrial weight/BW, g kg^−1^	0.33 ± 0.06	0.99 ± 0.22***	0.99 ± 0.19***	0.89 ± 0.26***	<.0001
Lung weight/BW, g kg^−1^	3.51 ± 0.37	4.96 ± 1.18***	5.01 ± 0.88***	4.28 ± 0.71*^#§^	<.0001
Liver weight/BW, g kg^−1^	34.2 ± 5.02	31.4 ± 5.55	32.6 ± 4.71	30.5 ± 6.96	.2
Kidney weight/BW, g kg^−1^	6.67 ± 0.64	5.52 ± 0.11***	5.8 ± 0.54***	5.78 ± 0.6***	<.0001
Heart failure score (0–7)	0.03	1.6***	1.5**	1.15*	.0008
Echocardiography: Left ventricle					
End‐diastolic dimension, mm	6.68 ± 0.64	12.52 ± 1.16***	12.81 ± 1.01***	11.5 ± 0.92***^##§§§^	<.0001
Posterior wall thickness, mm	2.65 ± 0.29	2.12 ± 0.25***	2.14 ± 0.2***	2 ± 0.33***	<.0001
Relative wall thickness	0.76 ± 0.12	0.33 ± 0.05***	0.32 ± 0.05***	0.34 ± 0.05***	<.0001
Fractional shortening, %	58.2 ± 4.8	38.3 ± 5.93***	38.8 ± 5.46***	42.7 ± 5.06***^#^	<.0001
Heart rate, min^−1^	457 ± 30.4	367 ± 36.9***	358 ± 45.3***	392 ± 39.2***^§^	<.0001
Stroke volume, mL	0.29 ± 0.08	1.47 ± 0.33***	1.63 ± 0.37***	1.27 ± 0.31***^§§§^	<.0001
Cardiac output, mL min^−1^	130 ± 31.1	534 ± 103***	570 ± 90.4***	493 ± 120***^§^	<.0001
Mitral regurgitation grade (1–4)	0.29	1.31**	1.22*	1.21*	.006
Echocardiography: Right ventricle					
RVD1, mm	3.61 ± 0.34	6.89 ± 1.27***	6.55 ± 0.71***	6.54 ± 1.31***	<.0001
RVD2, mm	3.49 ± 0.25	6.8 ± 1.23***	6.56 ± 0.81***	6.92 ± 1.43***	<.0001
RVD3, mm	9.7 ± 0.72	13.7 ± 1.45***	13.4 ± 0.98***	12.9 ± 1.4***	<.0001
RV diastolic area, mm^2^	31.8 ± 1.96	90.1 ± 21.2***	84.8 ± 12.9***	86.3 ± 21.1***	<.0001
Fractional area change, %	49.4 ± 4.1	41.8 ± 10.3**	42.2 ± 6.5*	43.9 ± 6.6	.006
TAPSE, mm	3.05 ± 0.21	3.94 ± 0.74***	4.29 ± 0.75***	3.96 ± 0.58***	<.0001
RV global strain, %	−9.3 ± 2.39	−13.2 ± 3.41**	−13.8 ± 4.43**	−12.6 ± 3.81*	.004
RV global strain rate, s^−1^	1.4 ± 0.31	1.97 ± 0.44***	2.11 ± 0.46***	1.89 ± 0.27**	<.0001
Tricuspid regurgitation grade (1–4)	0.43	1.25**	1.11	0.95	.01

*Note*: Values are expressed as means ± SD. *N* = 19 in sham/placebo group, *N* = 30 in ACF/placebo group, *N* = 20 in ACF/PDE5i group and *N* = 26 in ACF/ACEi group. Tukey post hoc test: *p < .05, **p < .01, ***p < .001 versus sham/placebo, ^#^ *p* < .05, ^##^ *p* < .01, ^###^ *p* < .001 versus ACF/placebo, ^§^ *p* < .05, ^§§^ *p* < .01, ^§§§^ *p* < .001 versus ACF/PDE5i.

Abbreviations: ACEi, angiotensin‐converting enzyme inhibitor; ACF, Rat model of aorto‐caval fistula; LV, left ventricle; PDE5i, phosphodiesterase‐5 inhibitor; RV, right ventricle; RVD1, right ventricular basal diameter at end‐diastole; RVD2, right ventricular mid diameter at end‐diastole; RVD3, right ventricular longitudinal diameter at end‐diastole; TAPSE, tricuspid annular plane systolic excursion.

### 3.2 Echocardiography

**FIGURE 1:**
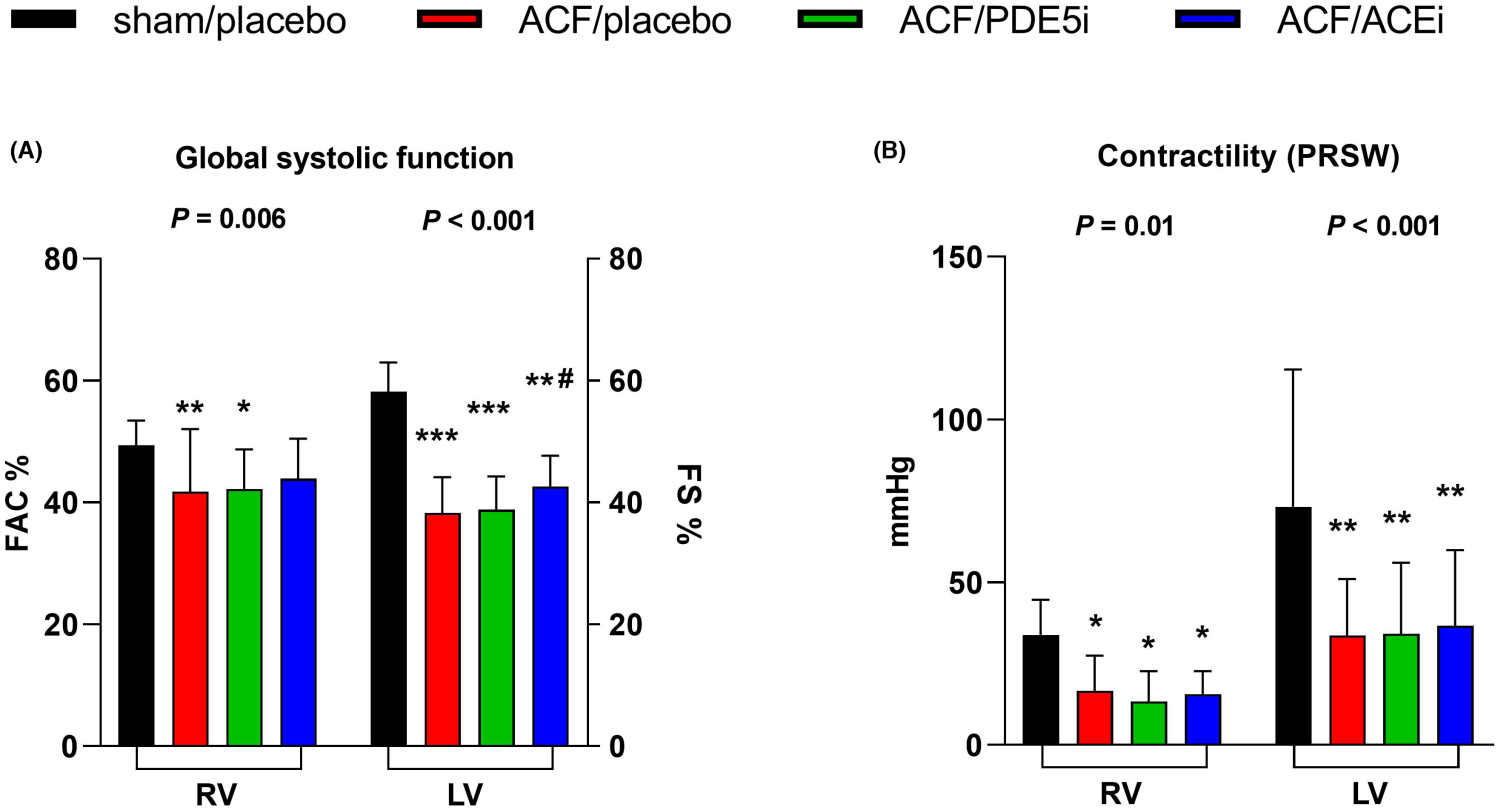
Effects of ACF, PDE5i, and ACEi on selected parameters of ventricular systolic function. (A) Global systolic function measured by echocardiography. (B) Contractility (PRSW) measured by pressure‐volume analysis. ACEi, angiotensin‐converting enzyme inhibitor; ACF, rat model of aorto‐caval fistula; LV, left ventricle; PDE5i, phosphodiesterase‐5 inhibitor; PRSW, preload recruitable stroke work; RV, right ventricle. *N* = 19–26 in each group. Data are presented as means ± SD. *p < .05, **p < .01, ***p < .001 versus sham/placebo, ^#^versus ACF/placebo.

Echocardiography (Table 1; Figure 1A) revealed LV dilatation, eccentric remodeling with relative wall thinning (relative wall thickness reduced by—57%, *p* < .001), and LV systolic dysfunction (LV fractional shortening [FS] reduced by—34%, *p* < .001) in ACF rats. Due to blood recirculation via the systemic shunt, cardiac output was increased 4.1‐fold (*p* < .001). The RV was also dilated and had depressed global systolic function (RV FAC reduced by—15%, *p* < .01). Regional echocardiographic parameters of RV function (TAPSE, global longitudinal strain) were affected by pronounced ventricular remodeling/dilatation and falsely augmented RV systolic function. ACEi limited LV remodeling and enhanced global LV systolic function (FS, +10%, *p* < .05) but PDE5i treatment did not show any beneficial effects.

### 3.3 Invasive hemodynamics and pressure‐volume analysis

In ACF, invasive hemodynamics (Table 2; Figure 1B) disclosed reduced systemic mean (−21 mmHg, *p* < .01) and diastolic blood pressure (−29 mmHg, *p* < .001), elevated end‐diastolic filling pressures (more in the LV, +5.6 mmHg, *p* < .001) and volumes (identically in both ventricles, LV 6.4‐fold, RV 5.9‐fold, *p* < .001), and increased ventricular wall stress (2.7‐fold, *p* < .001), as discussed previously.^5^ Maximal LV pressure was reduced (−33 mmHg, *p* < .001), but maximal RV pressure was raised (+11.8 mmHg, *p* < .001), and RV stroke work was increased relatively more (6.7‐fold, *p* < .001) compared to the LV (3.6‐fold, *p* < .001), due to additional pulmonary hypertension. RV load‐independent systolic function was similarly reduced compared to the LV (preload‐recruitable stroke work, LV: −54%, *p* < .01, RV: −51%, *p* < .05). ACEi lowered systemic blood pressure (−25 mmHg, *p* < .001), and reduced LV volumes (EDV −20%, ESV −39%, *p* < .01), wall stress (−19%, *p* < .05), and filling pressure (−2.4 mmHg, *p* < .05). PDE5i tended to reduce transpulmonary pressure gradient (probably due to vasodilatation in the pulmonary vascular bed), but it had a neutral effect on LV and RV parameters. Neither treatment affected the load‐independent systolic ventricular function of RV or LV (Figure 1B).

**TABLE 2:** Hemodynamic data from pressure‐volume analysis.

	Sham/placebo	ACF/placebo	ACF/PDE5i	ACF/ACEi	*p* (ANOVA)
Systemic circulation					
SBP, mmHg	143 ± 28.3	131 ± 21.2	124 ± 21.6*	106 ± 14***^###§^	<.0001
DBP, mmHg	113 ± 25.3	84 ± 14***	82 ± 15.9***	71 ± 12.9***^#^	<.0001
MBP, mmHg	128 ± 26.8	107 ± 16.7**	103 ± 18***	87 ± 13.4***^##^	<.0001
PP, mmHg	29.3 ± 5.6	47 ± 8.4***	42.7 ± 8.6***	35.2 ± 6.6^###§§^	<.0001
SVR, mmHg min mL^−1^	1.04 ± 0.26	0.21 ± 0.05***	0.19 ± 0.06***	0.19 ± 0.04***	<.0001
Left ventricle					
LV EDP, mmHg	6.35 ± 2.25	11.9 ± 4.14***	11.1 ± 3***	9.51 ± 2.16*^#^	<.0001
LV EDV, mL	0.31 ± 0.09	1.97 ± 0.54***	2.14 ± 0.54***	1.58 ± 0.39***^##§§§^	<.0001
LV mass/EDV, g mL^−1^	3.8 ± 0.91	1.1 ± 0.25***	1.01 ± 0.23***	1.17 ± 0.28***	<.0001
LV ESV, mL	0.02 ± 0.01	0.5 ± 0.28***	0.51 ± 0.21***	0.31 ± 0.12***^##§§^	<.0001
LV max pressure, mmHg	153 ± 26.8	120 ± 17.4***	120 ± 18.7***	105 ± 12.6***^#^	<.0001
LV max wall stress, mmHg mL g^−1^	43 ± 13.1	117 ± 34.5***	118 ± 24.1***	95 ± 20.2***^#§^	<.0001
Stroke work, mmHg mL	20.3 ± 6.17	73.1 ± 21.9***	83.9 ± 29.1***	70.3 ± 26.1***	<.0001
dP/dt max, mmHg s^−1^	10 405 ± 3965	8812 ± 3042	9800 ± 2445	8782 ± 2606	.3
PRSW, mmHg	73.2 ± 42.2	33.6 ± 17.4**	34.2 ± 21.8**	36.6 ± 23.3**	.0003
dP/dt min, mmHg s^−1^	−10 591 ± 3139	−5363 ± 1606***	−5939 ± 1651***	−5353 ± 1858***	<.0001
Tau, ms	11.2 ± 2.48	16.7 ± 3.8***	14.2 ± 3.43	13.4 ± 3.5^##^	<.0001
Right ventricle					
RV EDP, mmHg	4.04 ± 1.38	6.2 ± 1.95*	5.7 ± 2.59	5.03 ± 1.51	.03
RV EDV, mL	0.12 ± 0.01	0.71 ± 0.28***	0.64 ± 0.16***	0.7 ± 0.3***	<.0001
RV mass/EDV, g mL^−1^	2.37 ± 0.35	1.22 ± 0.31***	1.26 ± 0.27***	1.03 ± 0.24***	<.0001
RV ESV, mL	0.038 ± 0.005	0.033 ± 0.2***	0.274 ± 0.09***	0.287 ± 0.168***	<.0001
RV max pressure, mmHg	34.1 ± 4.9	45.9 ± 6.39***	44.5 ± 3.44***	42.6 ± 6.16***	<.0001
RV max pressure—LVEDP gradient, mmHg	27.9 ± 6.2	35.1 ± 5.31**	33.3 ± 2.72	33.9 ± 6.83*	.01
RV max wall stress, mmHg mL g^−1^	15.2 ± 3.15	35.8 ± 9.24***	35.8 ± 8.26***	43.8 ± 16***	<.0001
Stroke work, mmHg mL	1.09 ± 0.14	7.33 ± 2.46***	7.65 ± 2.29***	7.09 ± 2.88***	.0004
dP/dt max, mmHg s^−1^	2222 ± 788	2908 ± 767	2700 ± 446	2689 ± 1079	.2
PRSW, mmHg	33.9 ± 10.77	16.7 ± 10.79*	13.4 ± 9.32*	15.6 ± 7.02*	.01
dP/dt min, mmHg s^−1^	−1621 ± 297	−1610 ± 442	−1709 ± 245	−1603 ± 493	.9
Tau, ms	20.7 ± 12.79	20.7 ± 9.54	16.3 ± 4.65	20.7 ± 14.8	.7

*Note*: Values are expressed as means ± SD. *N* = 19 in sham/placebo group, *N* = 30 in ACF/placebo group, *N* = 20 in ACF/PDE5i group and *N* = 26 in ACF/ACEi group. Tukey post hoc test: *p < .05, **p < .01, ***p < .001 versus sham/placebo, ^#^ *p* < .05, ^##^ *p* < .01, ^###^ *p* < .001 versus ACF/placebo, ^§^ *p* < .05, ^§§^ *p* < .01, ^§§§^ *p* < .001 versus ACF/PDE5i.

Abbreviations: ACEi, angiotensin‐converting enzyme inhibitor; ACF, rat model of aorto‐caval fistula; DBP, diastolic blood pressure; dP/dt max, maximum ventricular pressure over time; dP/dt min, minimum ventricular pressure over time; EDP, ventricular end‐diastolic pressure; EDV, ventricular end‐diastolic volume; ESV, ventricular end‐systolic volume; LV, left ventricle; MBP, mean blood pressure; PDE5i, phosphodiesterase‐5 inhibitor; PP, pulse pressure; PRSW, preload recruitable stroke work; RV, right ventricle; SBP, systolic blood pressure; SVR, systemic vascular resistance; Tau, ventricular diastolic time constant.

### 3.4 Myocardial gene expression analysis

**FIGURE 2:**
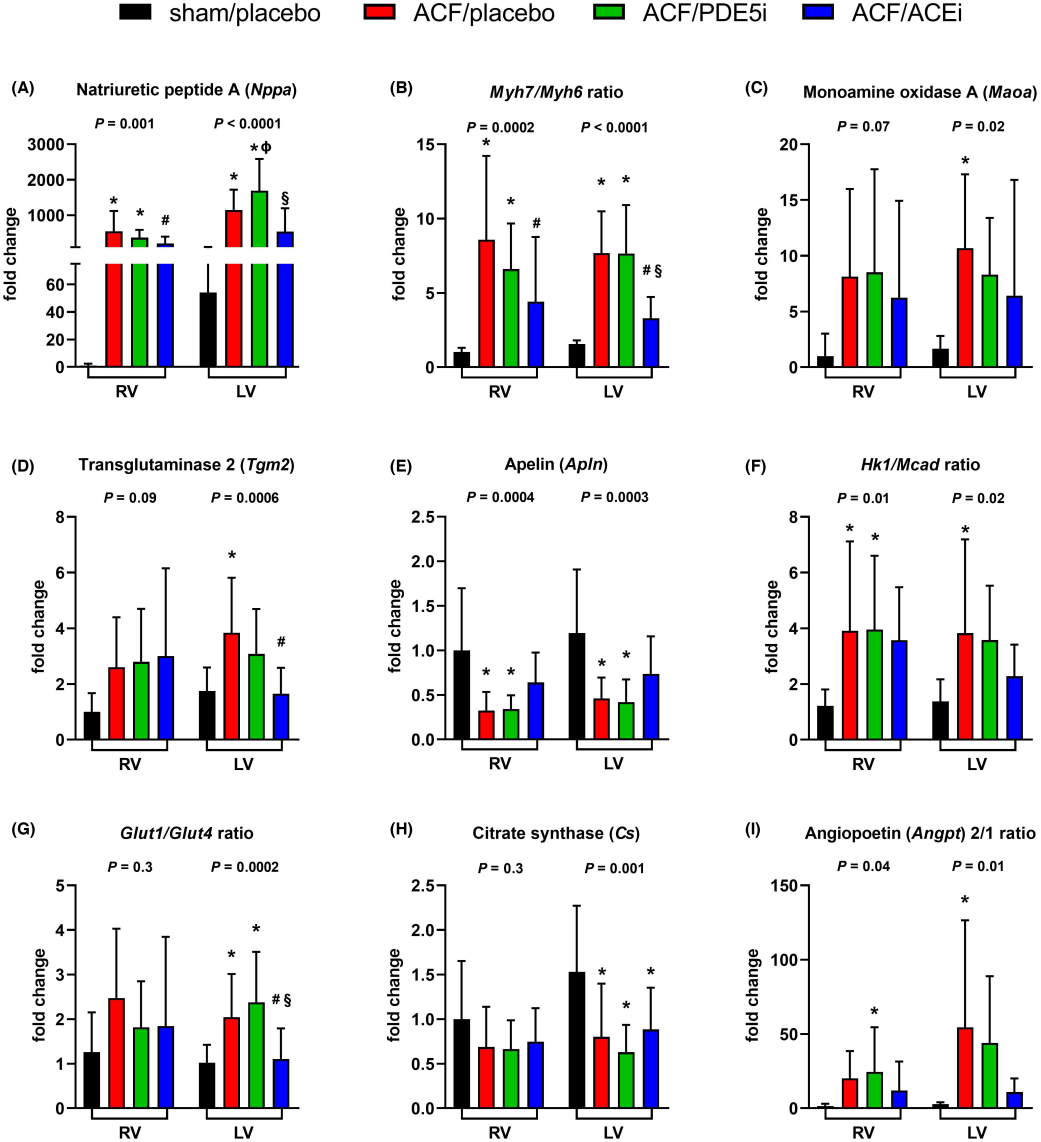
Effects of ACF, PDE5i, and ACEi on gene expression of selected cardiac markers of stress and metabolism. mRNA expression analysis of selected genes that reflect myocardial stress, substrate metabolism, and bioenergetics. (A) Natriuretic peptide A (*Nppa*). (B) *Myh7/Myh6* ratio. (C) Monoamine oxidase A (*Maoa*). (D) Transglutaminase 2 (*Tgm2*). (E) Apelin (*Apln*). (F) *Hk1/Mcad* ratio. (G) *Glut1/Glut4* ratio. (H) Citrate synthase (*Cs*). (I) Angiopoetin (*Angpt*) 2/1 ratio. Data are presented as means ± SD. The changes are normalized to sham/placebo RV. *N* = 12–13 in each group. ACF, rat model of aorto‐caval fistula; PDE5i, phosphodiesterase‐5 inhibitor; ACEi, angiotensin‐converting enzyme inhibitor; RV, right ventricle; LV, left ventricle; *Myh7/Myh6* ratio, myosin heavy chain isotype 7 to 6 ratio; *Hk1/Mcad* ratio, ratio of glycolytic enzyme hexokinase to medium‐chain acyl‐CoA dehydrogenase; *Glut1/Glut4* ratio, glucose transporter 1/4 ratio. *p < .05 versus sham/placebo, ^#^versus ACF/placebo, ^§^versus ACF/PDE5i, ^ϕ^versus RV of the same group.

mRNA expression analysis of selected genes by qPCR (Figure 2; Supporting Information 2, for a full list see materials and methods section) revealed ACF‐induced upregulation of several genes associated with myocardial stress, especially natriuretic peptide A (*Nppa*, RV 549‐fold, LV 21‐fold, *p* < .05). ACF rats exhibited elevated myosin heavy chain isotype 7 to 6 ratio (*Myh7/Myh6*, RV 8.4‐fold, LV 4.9‐fold, *p* < .05), angiopoietin 2 to 1 ratio (*Angpt2/1*, LV 19.8‐fold, *p* < .05), downregulated apelin (*Apln*, RV 0.7‐fold, LV 0.6‐fold, *p* < .05) and citrate synthase (*Cs*, LV 0.5‐fold, *p <* .05), and upregulated monoamine oxidase‐A (*Maoa*, LV 9.2‐fold, *p <* .05) and transglutaminase‐2 (*Tgm2*, LV 2.2‐fold, *p <* .05), genes that were previously linked to ACF.^38^ There was an apparent metabolic shift from fatty acid oxidation to glycolysis reflected by an elevated ratio (RV 3.2‐fold, LV 2.8‐fold, *p <* .05) of glycolytic enzyme hexokinase 1 (*Hk1*) to medium‐chain acyl‐CoA dehydrogenase (*Mcad*) and an increased ratio of glucose transporters *Glut1/Glut4* (LV 2‐fold, *p <* .05). Gene expression changes caused by ACF‐induced VO were similar in both ventricles and were partially reversed by ACEi treatment. ACEi downregulated *Nppa* (RV 0.6‐fold, *p <* .05) and *Myh7/Myh6* ratio (RV 0.5‐fold, LV 0.6‐fold, *p <* .05). There was also a trend to downregulated *Maoa* (RV 0.2‐fold, LV 0.4‐fold) and reduced *Hk1/Mcad* ratio (LV 0.4‐fold). ACEi downregulated *Tgm2* (LV 0.6‐fold, *p* < .05) and reduced *Glut1/Glut4* (LV 0.5‐fold, *p* < .05) ratio in ACF LV. PDE5i had no beneficial effects. ACF or the treatments did not alter the expression of genes of cGMP‐dependent signaling pathway, that is, NO synthase 1–3 (*Nos 1–3*), natriuretic peptide receptor 1 and 2 (*Npr1* and *Npr2*), soluble guanylate cyclase (*Gucy1a3*), cGMP‐dependent protein kinase (*Pkg*), phosphodiesterase 5 (*Pde5a*), and phosphodiesterase 9 (*Pde9a*, Supporting Information 2).

### 3.5 Renal hemodynamics and excretory function

**FIGURE 3:**
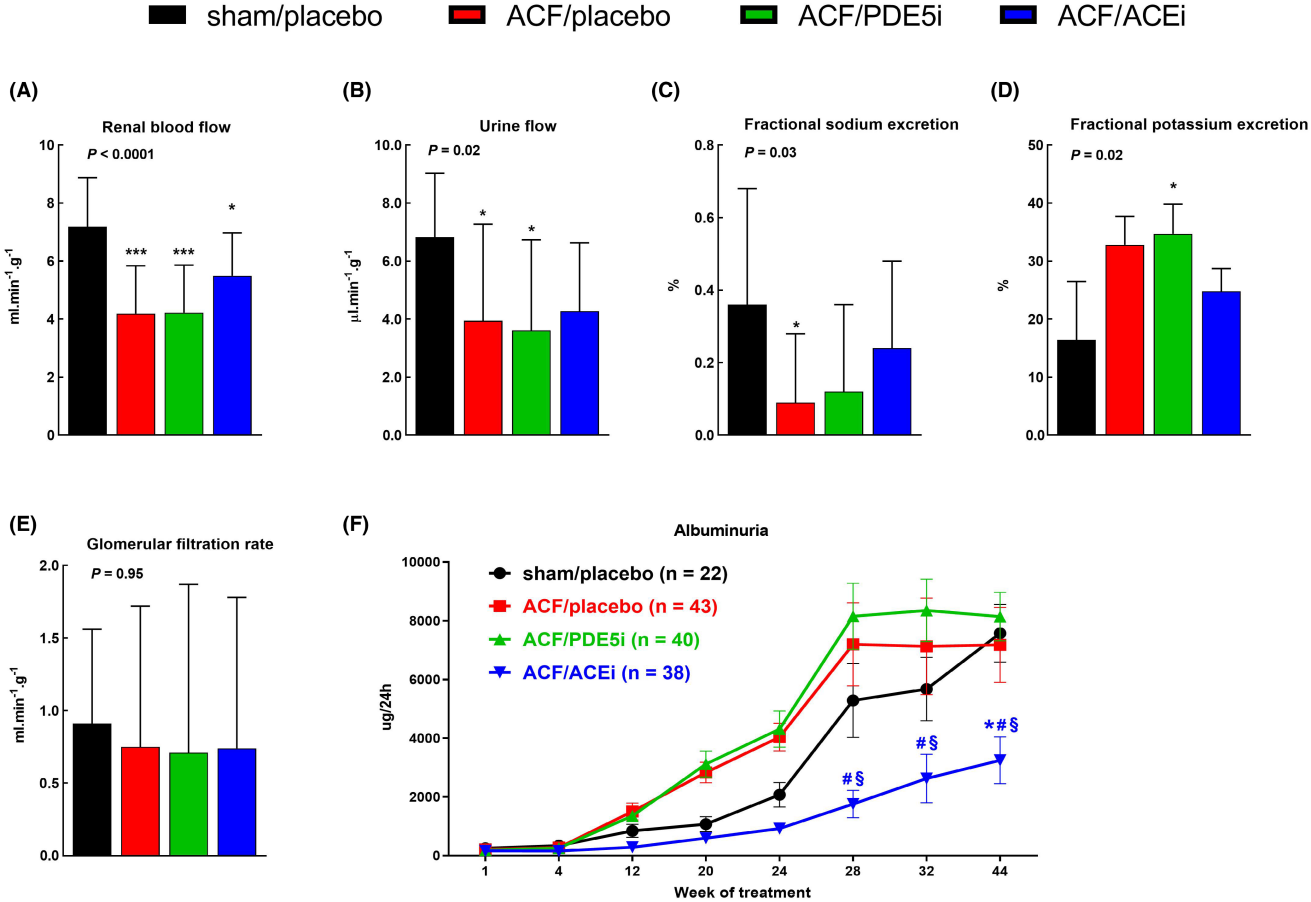
Effects of ACF, PDE5i, and ACEi on renal hemodynamics, excretory function, and albuminuria. (A) Renal blood flow. (B) Urine flow. (C) Fractional sodium excretion. (D) Fractional potassium excretion. (E) Glomerular filtration rate. (F) Albuminuria. Data in A–E are expressed per gram of wet kidney weight and presented as means ± SD. ACEi, angiotensin‐converting enzyme inhibitor; ACF, rat model of aorto‐caval fistula; PDE5i, phosphodiesterase‐5 inhibitor. *N* = 13–16 in each group. *p < .05, ***p < .001 versus sham/placebo, ^#^versus ACF/placebo, ^§^versus ACF/PDE5i.

In comparison with healthy controls, ACF animals exhibited significantly lower renal blood flow (Figure 3A, 4.19 ± 1.65 vs. 7.18 ± 1.69, *p* < .001), decreased diuresis (Figure 3B, urine flow 3.94 ± 3.33 vs. 6.83 ± 2.2, *p* < .05), and decreased fractional sodium excretion (Figure 3C, 0.09 ± 0.19 vs. 0.36 ± 0.32, *p* < .05). There was a trend toward higher fractional potassium excretion (32.8 ± 4.9 vs. 16.4 ± 10.1), probably due to secondary hyperaldosteronism (Figure 3D). ACEi tended to raise renal blood flow (5.49 ± 1.48 vs. 4.19 ± 1.65), urine flow (4.27 ± 2.36 vs. 3.94 ± 3.33), and fractional sodium excretion (0.24 ± 0.24 vs. 0.09 ± 0.19) and to decrease fractional potassium excretion (24.8 ± 3.9 vs. 32.8 ± 4.9); however, the differences were not statistically significant. PDE5i did not cause any beneficial effects. Glomerular filtration rate was not significantly different among groups, though it tended to be lower in ACF animals compared to sham‐operated rats (Figure 3E, 0.75 ± 0.97 vs. 0.91 ± 0.65).

### 3.6 Albuminuria

Aging of both ACF and sham‐operated animals was accompanied by gradually increasing albuminuria, which was effectively suppressed with ACEi (Figure 3F). At the end of the study, albuminuria of ACEi‐treated ACF animals was even lower than in healthy controls (3000 ± 2000 μg/24 h vs. 8000 ± 3000 μg/24 h, *p* = .001). PDE5i had no beneficial effect.

### 3.7 Survival

**FIGURE 4:**
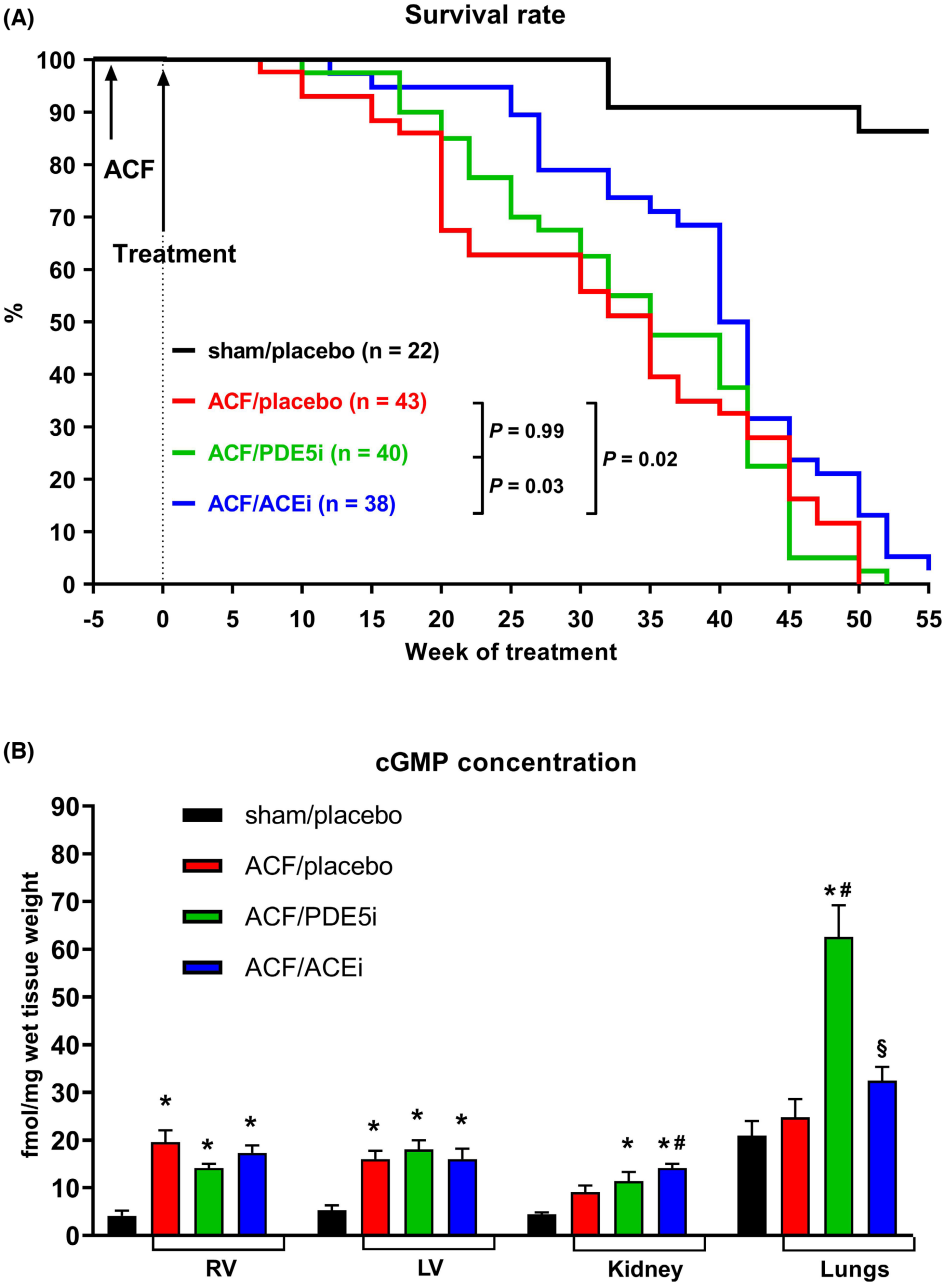
Effects of ACF, PDE5i, and ACEi on survival rate and cGMP tissue levels. (A) Survival rates. (B) cGMP tissue levels in the right and left ventricle, kidney, and lungs. Data in (B) are expressed as fmol cGMP per milligram of wet tissue weight and presented as means ± SD. *N* = 8 in each group. ACEi, angiotensin‐converting enzyme inhibitor; ACF, rat model of aorto‐caval fistula; cGMP, cyclic guanosine monophosphate; LV, left ventricle; PDE5i, phosphodiesterase‐5 inhibitor; RV, right ventricle. *p < .05 versus sham/placebo, ^#^versus ACF/placebo, ^§^versus ACF/PDE5i.

At the end of the study (55 weeks after induction of ACF), all untreated ACF animals were dead. Median survival in ACF/placebo group was 35 weeks, identical to that in PDE5i‐treated rats (Figure 4A). On the contrary, ACEi significantly improved the survival rate (median survival in ACF/ACEi group was 41 weeks, *p* = .02 compared to untreated ACF rats). The beneficial influence of ACEi was most apparent in the first weeks after initiation of treatment; however, ACEi‐treated ACF rats started to die rapidly after week 40 and only 2 of them survived till the end of the study (the survival rate at 55 weeks after induction of ACF was 5%). We observed 3 deaths (14%) in the sham‐operated control group, probably due to old age.

### 3.8 cGMP tissue levels

Myocardial cGMP levels were increased in ACF RV (19.6 ± 7 vs. 4.1 ± 3.1, *p* < .05) and LV (16 ± 5 vs. 5.3 ± 3, *p* < .05), probably due to stimulation of NP‐receptor‐associated particulate guanylate cyclases by elevated levels of natriuretic peptides. PDE5i raised cGMP concentration in the lungs (63 ± 19 vs. 21 ± 8.6, *p* < .05), however, not in the myocardium and the kidney (Figure 4B).

## 4 DISCUSSION

The study describes the impact of long‐term PDE5i and ACEi treatments in a rat HF model due to chronic VO induced by ACF. The main finding is that long‐term therapy with PDE5i sildenafil does not improve the survival rate, and cardiac and renal function of animals with ACF. There were no relevant differences in the LV and RV responses to long‐term PDE5i treatment. Finally, long‐term ACEi therapy improved survival of ACF rats despite relatively small effects on cardiac remodeling, myocardial gene expression, and renal hemodynamics. The results indicate that cGMP‐dependent signaling pathway does not play a major role in response to ACF, while supporting the role of ACEi in treating HF due to VO.

While the effects of PDE5i on hypertrophy and cardiac dysfunction were repeatedly studied in pressure overloaded heart,^13^, ^14^, ^15^ there is a paucity of studies that examined the effects on volume overloaded heart. To our knowledge, this is the first such a complex evaluation of long‐term PDE5i treatment in VO that leads to overt HF. In the present study, employing comprehensive analysis of both RV and LV structure and load‐independent function, myocardial gene expression, renal function assessment, and survival, we have not demonstrated any benefit of PDE5i after 20 weeks of treatment. A lack of protective effect of PDE5i treatment on ACF RV was described previously,^14^ however, that study was limited to RV assessment and sildenafil was administered for 4 weeks only.

In another experimental VO model, Kim et al. demonstrated a significant attenuation of adverse LV remodeling and improvement of exercise capacity following a 4‐month sildenafil treatment.^39^ However, the rats in that study exhibited only a compensated LV hypertrophy rather than true HF, without increased chamber filing pressures. Endothelial NO synthase (NO synthase 3) was downregulated in untreated mitral regurgitation and it was increased by sildenafil. In our study, the expression of NO synthase 3 had a trend to decrease in ACF, with no effect of PDE5i treatment, which may explain the discrepant results between the two different models. Eskesen et al. observed improved LV function and less LV remodeling after PDE5i in a rat HF model of aortic regurgitation, which is however a model of combined pressure and VO, so the beneficial effects of PDE5i could be explainable by the reduction of pressure component.^40^

There may be several possible explanations for the lack of efficacy of PDE5i treatment in preventing hypertrophy and failure of VO heart. First, the administered dose of sildenafil could be insufficient to suppress PDE5 activity. However, this is improbable, as this dose is well above that previously shown to raise plasma cGMP levels in rats,^16^ and we measured elevated cGMP levels in the lungs of sildenafil‐treated rats, which confirmed the effectiveness of treatment. Second, cGMP synthesis in ACF myocardium could be insufficient. We observed increased cGMP concentrations both in ACF RV and LV, likely due to stimulation of particulate guanylate cyclase by elevated levels of natriuretic peptides. However, subcellular cGMP pool compartmentalization may also play a role, because PDE5i was demonstrated to exclusively increase the cGMP generated by soluble guanylate cyclase in response to NO, while the cGMP pool derived from natriuretic peptides‐stimulated particulate guanylate cyclase was not affected in rat cardiomyocytes.^41^ The third possible explanation could be the cGMP degradation by phosphodiesterases other than PDE5, particularly PDE9.^42^ However, we did not observe *Pde9a* gene upregulation in ACF hearts. Finally, the cGMP‐dependent signaling pathway may not be involved in the myocardial response to ACF‐induced VO, and our gene expression data support this mechanism. ACF did not alter the expression of genes of the cGMP‐dependent signaling pathway, arguing against a substantial role of this pathway in response to VO, at least in the ACF model of advanced HF. In addition, both ACF LV and RV exhibited eccentric hypertrophy, decreased contractility, and upregulation of genes connected with myocardial stress (*Nppa, Myh7/Myh6 ratio*), and metabolic switch from fatty acid oxidation to glycolysis (increased *Glut1/Glut4* and *Hk1/Mcad ratio*). The presented data provide no evidence for the heart chamber‐specific responsiveness to long‐term PDE5i.

Among other phosphodiesterases, PDE5 is also highly expressed in the kidney and was proposed to contribute to the blunted renal response to elevated levels of endogenous natriuretic peptides in advanced HF.^43^, ^44^ In the present study, we have not seen any beneficial effect of PDE5i treatment on renal hemodynamics and excretory function. Several experimental studies in dogs with overt HF induced by rapid ventricular pacing demonstrated improvement of renal function following administration of PDE5i either alone^43^, ^45^ or in combination with exogenous brain natriuretic peptide^46^ or with PDE9 inhibition.^47^ In all the above studies, phosphodiesterase inhibition was associated with increased renal cGMP concentrations. However, in our study, sildenafil failed to significantly raise renal cGMP level, although it tended to be higher in sildenafil‐treated compared to placebo‐treated rats. Based on our results, it is likely that renal PDE5 activity is not upregulated by ACF‐induced VO.

A significant amount of PDE5 is present in the lung tissue, and inhibiting PDE5 leads to a decrease in pulmonary vascular resistance both in experimental models and also in patients with HF and pulmonary hypertension.^48^, ^49^, ^50^ Based on our cGMP data, upregulation of PDE5 is likely to occur in the lungs of ACF rats, as PDE5i‐treated animals have highly increased lung cGMP concentration.^50^

Finally, our study demonstrated that long‐term ACEi improves the survival of ACF rats despite relatively small effects on cardiac remodeling, myocardial gene expression, and renal hemodynamics. ACEi treatment decreased systemic blood pressure, lowered LV maximal and filling pressures, reduced LV wall stress and LV volumes, attenuated albuminuria, diminished pulmonary congestion, and reduced body weight of ACF animals, likely due to alleviating congestion. However, treatment with ACEi had no effect on RV parameters, did not enhance load‐independent LV systolic function, and did not significantly raise renal blood flow, urine flow, and fractional sodium excretion, confirming our previous observations.^51^, ^52^ The exact mechanisms of how ACEi protects against increased HF mortality due to ACF‐induced VO, despite modest effects on cardiorenal function, deserve further investigation. Jarkovska et al. suggested the anti‐arrhythmic effect of ACEi in ACF rats.^53^

The present study has several limitations. Only the resting hemodynamics was tested, because of technical reasons we did not perform preload changing maneuvers (vena cava balloon inflation), and therefore we cannot report arterial and ventricular elastance values. We tested the changes in myocardial gene expression only, but we did not perform proteomic analysis. We also did not measure protein kinase G activity and plasma and renal angiotensin levels as markers of PDE5i and ACEi efficacy, respectively. This study does not include a sham/control group treated with PDE5i or ACEi, which could add to a deeper understanding of individual treatments, but our question was narrowed down to the effect of treatment in rats with HF, and thus the primary focus was on translational research. Despite the known differences between sexes in various HF models, our current study has deliberately focused on only one gender. This approach stems from our previous investigation, in which no distinctions between sexes were observed in relation to the ACF model within the HanSD strain.^54^

In conclusion, the study shows that PDE5i does not improve survival rate and does not change the cardiac and renal function of ACF rats with advanced HF. The cGMP‐dependent signaling pathway does not seem to play a major role in response to ACF‐induced VO. In contrast, mortality in the ACF model was reduced by ACEi treatment.

## AUTHOR CONTRIBUTIONS

Participated in research design: T.T. and V.M. Conducted experiments: T.T., M.K., P.S., L.K., J.T., V.S., M.K., and J.N. Performed data analysis: T.T., M.M., M.K., P.S., and J.N. Wrote or contributed to the writing of the manuscript: T.T., M.M., M.K., P.S., L.K., J.T., V.S., M.K., J.N., and V.M.

## FUNDING INFORMATION

Supported by Ministry of Health of the Czech Republic, grant no. NU22‐02‐00161, NU20‐02‐00052; Grant Agency of Charles University (GAUK), grant no. 304121; All rights reserved. Project National Institute for Research of Metabolic and Cardiovascular Diseases (Programme EXCELES, Project no. LX22NPO5104), Funded by the European Union—Next Generation EU.

## CONFLICT OF INTEREST STATEMENT

The authors declare that they have no competing interests.

## ETHICS STATEMENT

This study was performed in accordance with the NIH Guide for the Care and Use of Laboratory Animals (NIH Publication No. 85‐23, 1996) and Animal protection laws of the Czech Republic (311/1997) and was approved by the Ethic Committee of IKEM.

## Supporting information


Appendix S1.

## Data Availability

The data that support the findings of this study are available on request from the corresponding author.
