# Supplementary material for: The impact of phosphodiesterase‐5 inhibition or angiotensin‐converting enzyme inhibition on right and left ventricular remodeling in heart failure due to chronic volume overload
Source: Pharmacol Res Perspect. 2024 Jan 29;12(1):e1172. doi: 10.1002/prp2.1172 (PMC10823410; doi:10.1002/prp2.1172)
Supplement: Supplementary file 1 — Appendix S1. [file PRP2-12-e1172-s001.zip › prp21172-sup-0003-Supportinginformation3.docx]

**Supporting information 1: Effects of ACF, PDE5i and ACEi on body weight.** Data are presented as means ± SD. N=8 in each group. Rat model of aorto-caval fistula (ACF); phosphodiesterase-5 inhibitor (PDE5i); angiotensin-converting enzyme inhibitor (ACEi).

**Supporting information 2: Effects of ACF, PDE5i and ACEi on myocardial expression of genes of cGMP signaling pathway.** Gene mRNA expression analysis of genes of cGMP-dependent signaling pathway. Data are presented as means ± SD. The changes are normalized to sham/placebo RV. N=12-13 in each group. Rat model of aorto-caval fistula (ACF); phosphodiesterase-5 inhibitor (PDE5i); angiotensin-converting enzyme inhibitor (ACEi); right ventricle (RV), left ventricle (LV).
