# Supplementary figures and images for: The impact of phosphodiesterase‐5 inhibition or angiotensin‐converting enzyme inhibition on right and left ventricular remodeling in heart failure due to chronic volume overload
Source: Pharmacol Res Perspect. 2024 Jan 29;12(1):e1172. doi: 10.1002/prp2.1172 (PMC10823410; doi:10.1002/prp2.1172)

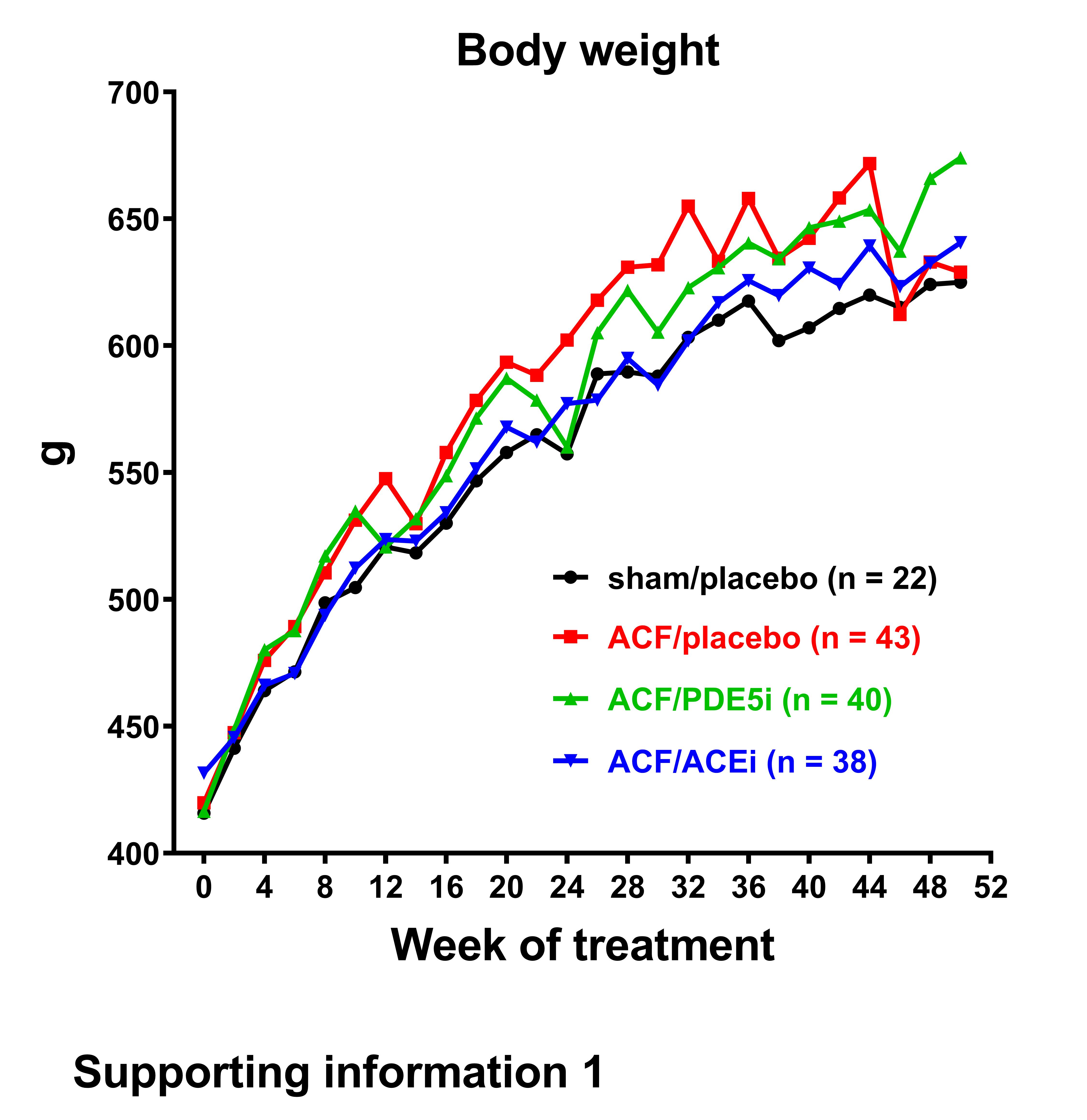

Supplement: Supplementary file 1 — Appendix S1. [file PRP2-12-e1172-s001.zip › prp21172-sup-0001-Supportinginformation1.jpg]

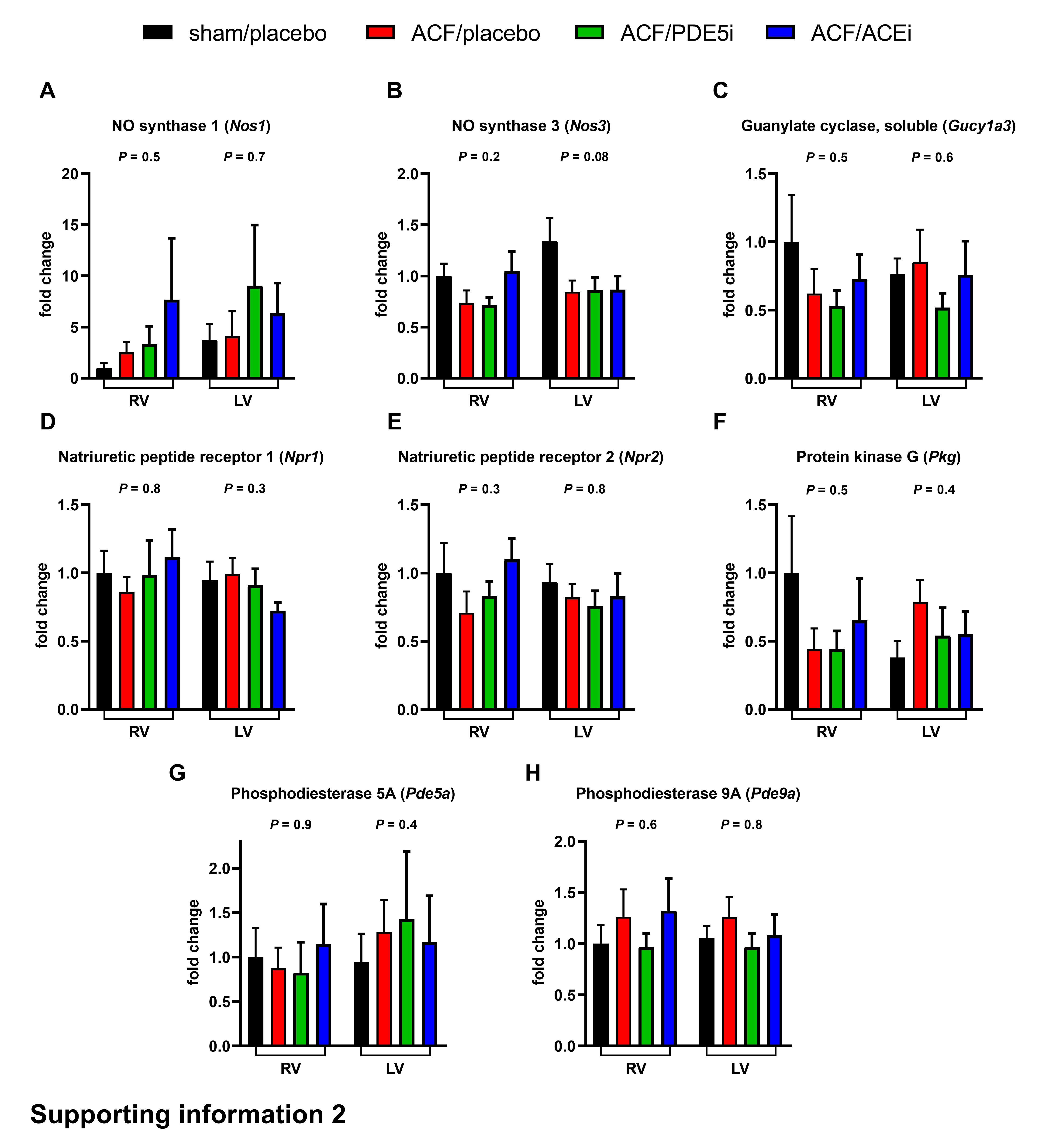

Supplement: Supplementary file 1 — Appendix S1. [file PRP2-12-e1172-s001.zip › prp21172-sup-0002-Supportinginformation2.jpg]
